# Personalized Remote Patient Management in Heart Failure: From Implanted Hemodynamic Monitoring Toward an Artificial Intelligence-Supported Adaptive Care Cycle

**DOI:** 10.1007/s11897-026-00783-8

**Published:** 2026-10-19

**Authors:** Christiane E. Angermann, Folkert W. Asselbergs, Georg Ertl, Stefan Störk

**Affiliations:** 1 Comprehensive Heart Failure Centre, University and University Hospital Würzburg, Germany, and Department of Medicine 1, Cardiology, University Hospital Würzburg, Am Schwarzenberg 15, 97078 Würzburg, Germany; 2 Department of Cardiology, Amsterdam Cardiovascular Sciences, Amsterdam University Medical Centre, University of Amsterdam, Amsterdam, the Netherlands; 3 Institute of Health Informatics, University College London, London, UK; 4 The National Institute for Health Research University College London Hospitals Biomedical Research Centre, University College London, London, UK; 5 Comprehensive Heart Failure Centre, Department of Clinical Research & Epidemiology, Würzburg, Germany

**Keywords:** Heart failure, Artificial intelligence, Pulmonary artery pressure, Implanted hemodynamic monitoring, Remote patient management, Quality of care

## Abstract

**Purpose of Review:**

Implanted pulmonary artery pressure (PAP) monitoring provides an early window on imminent worsening heart failure (HF). Randomized trials demonstrated that PAP-guided management significantly reduces HF hospitalizations, but substantial residual risk persists despite recent major advances in HF pharmacotherapy. We review lessons from PAP-guided remote patient management and examine how artificial intelligence (AI) could support more individualized eligibility assessment and multimodal longitudinal surveillance within a patient-centered adaptive care cycle to further improve clinical outcomes.

**Recent Findings:**

HF is increasingly recognized as a systemic disease characterized, across HF phenotypes, by multiple cardiac and non-cardiac comorbidities. CHAMPION, GUIDE-HF, and MONITOR-HF did not systematically incorporate comorbidity-related risk into patient selection, while PAP-guided treatment focused predominantly on diuretic adjustment, and monitoring strategies were not adapted to individual disease trajectories.

**Summary:**

AI could help integrate multidimensional phenotyping to estimate individual risk and expected benefit pre-implant, use comprehensive longitudinal monitoring data to support personalized, actionable clinical decision-making, and enable dynamic adaptation of monitoring intensity tailored to the disease trajectory. This may improve clinical outcomes, reduce healthcare professional workload, enhance patient experience and treatment adherence, and increase cost-effectiveness.

Pulmonary artery pressure (PAP) rises before overt clinical congestion, providing an opportunity to intervene before worsening heart failure (HF) necessitates hospital admission [1]. Randomized trials have established that PAP-guided management reduces HF hospitalizations (HFH) [2–5], and implanted hemodynamic monitoring is now moving toward reimbursed routine care. In the Netherlands, PAP-guided monitoring has been included in the basic health-insurance package since May 2024 for selected patients with NYHA class III HF symptoms and a previous HFH [6]. Germany is now moving in the same direction: the Federal Joint Committee (G-BA) has concluded that the benefit and medical necessity of implanted PAP monitoring in NYHA class III HF are established and has initiated the consultation process toward its inclusion in reimbursed care [7]. This transition provides an important impetus for considering how implanted PAP monitoring should evolve as it enters broader clinical practice. Wider implementation raises two questions not resolved by previous trials: who is most likely to derive meaningful benefit from a PAP-monitoring device, and how can increasing volumes of longitudinal data be translated into timely, actionable care without creating an unsustainable monitoring burden? This Spotlight explores how AI may support individualized benefit prediction, efficient integration and interpretation of longitudinal data, and the evolution of PAP-guided remote patient management toward an adaptive, patient-centered care cycle.

## Implanted Hemodynamic Monitoring: Lessons from the Past

CHAMPION established more than a decade ago that PAP-guided management can reduce HFH in high-risk populations, with GUIDE-HF and MONITOR-HF subsequently extending this evidence across a broader HF spectrum and different eras of pharmacotherapy [2–5]. Despite differences in design and context, effects on HFH were remarkably consistent, and a meta-analysis of the three trials demonstrated an approximately 30% reduction in total HFH, while all-cause mortality was not significantly reduced [8]. Importantly, however, a subsequent patient-level meta-analysis of CHAMPION, GUIDE-HF and LAPTOP-HF in patients with HF with reduced ejection fraction reported reductions in both HFH and all-cause mortality, with the hospitalization benefit occurring early and the survival benefit emerging only after the first year of monitoring [9]. Wireless PAP monitoring now has a Class IIa, Level B1, recommendation in the 2026 ESC HF guidelines [10], based on the CHAMPION, GUIDE-HF and MONITOR-HF trials, to reduce HFH in selected symptomatic patients.

**Fig. 1:**
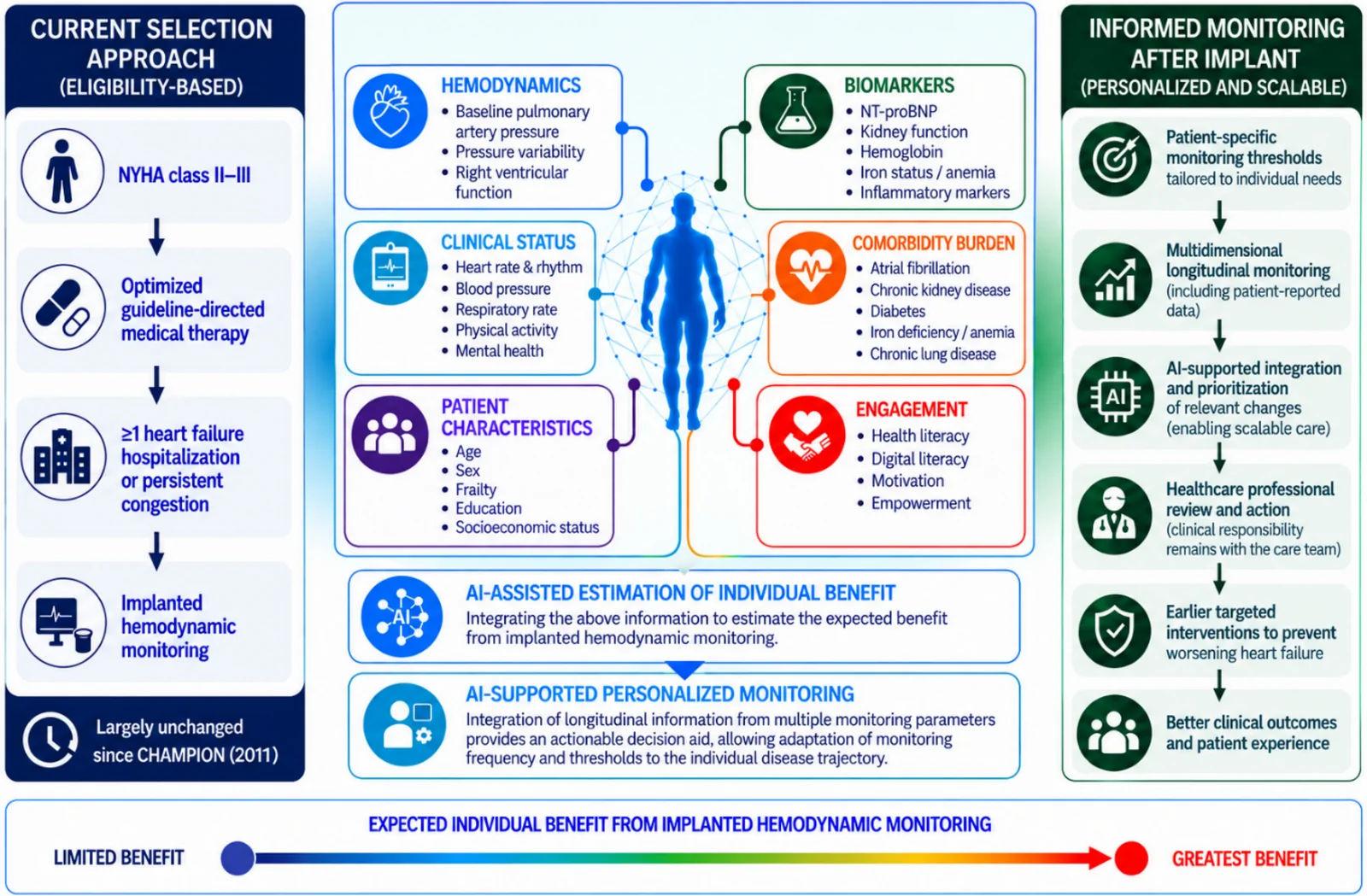
**Conceptual framework for AI-supported personalization of implanted hemodynamic monitoring in heart failure**. Current patient selection for implanted hemodynamic monitoring relies largely on the inclusion criteria of previous randomized trials evaluating PAP-guided remote patient management, such as NYHA functional class, optimized guideline-directed medical therapy, previous heart failure hospitalization and/or persistent congestion (left). A multidimensional patient profiling approach could integrate hemodynamics, clinical status, patient characteristics, biomarkers, comorbidity burden, and patient engagement to support AI-assisted assessment of eligibility, individual attainable benefit from implanted monitoring and pre- and peri-implant care requirements (center). Corresponding data could subsequently also inform personalized post-implant surveillance, including patient-specific monitoring thresholds and dynamic adaptation of monitoring intensity to the individual disease trajectory. AI-supported integration and prioritization of longitudinal information could facilitate scalable remote management, with healthcare professionals retaining clinical responsibility for review and action (right). The lower continuum illustrates that expected benefit may vary substantially among patients eligible based on current criteria. AI, artificial intelligence; HF, heart failure; NT-proBNP, N-terminal pro-B-type natriuretic peptide; NYHA, New York Heart Association; PAP, pulmonary artery pressure

For more than a decade, the approach to patient selection and subsequent PAP-guided management has changed little. Eligibility has continued to rely on trial-derived criteria such as functional class, previous HFH and/or elevated natriuretic peptides, while therapeutic decisions have been guided mainly by PAP changes (Fig. 1, left). Treatment adaptation focused on decongestion, with diuretic adjustment the most frequent intervention, although vasodilator and neurohormonal therapies were also modified [11, 12]. PAP monitoring detected hemodynamic deterioration sufficiently early to guide therapeutic interventions that prevented HFH. More recent patient-level meta-analyses further demonstrated that both absolute PAP levels and their longitudinal changes are independently associated with subsequent HFH and mortality risk [13, 14]. Nevertheless, substantial residual morbidity and mortality remained despite concurrent remarkable improvements in HF pharmacotherapy. The delayed survival benefit observed with longer-term hemodynamic monitoring is intriguing [9], suggesting that prevention of repeat worsening HF may require prolonged periods of hemodynamic stability before translating into sustainable prognostic benefit, depending also on disease stage and competing risks. Moreover, neither patient eligibility assessment nor PAP-guided remote care strategies systematically incorporated the growing appreciation of HF as a systemic disease or addressed, how much of an individual patient’s risk is actually modifiable by hemodynamic-guided care.

Experience with CIED-based monitoring provides another important lesson. In IN-TIME, daily multiparametric monitoring improved clinical outcomes, including mortality, but required intensive clinical workflows with daily review, rapid interpretation and timely patient contact [15, 16]. Effective remote management therefore depended not only on detecting deterioration, but on translating complex monitoring data into timely clinical action. Such healthcare professional-intensive workflows may become increasingly difficult to sustain as implanted monitoring enters reimbursed clinical care. AI could help clinical teams prioritize actionable information and reduce repetitive review, but workflow efficiency should be evaluated prospectively rather than assumed as an inherent benefit.

## Beyond Traditional Eligibility Assessment: Who Benefits most from Implanted Monitoring?

Established selection criteria identify patients at increased risk of adverse clinical outcomes, but individuals meeting the same criteria may differ substantially in HF phenotype, comorbidity profile, disease trajectory and the individual benefit to be expected from PAP-guided remote patient management.

Comprehensive phenotyping should therefore integrate hemodynamics and cardiac function with factors such as symptom trajectory, vital status, laboratory biomarkers, comorbidity burden, diuretic responsiveness and tolerability of other foundational medical treatment, demographic and frailty characteristics, as well as psychosocial factors and health and digital literacy, which are all relevant to successful remote management (Fig. 1, center) [17, 18]. Such multidimensional pre-implant profiling could enable a more accurate estimate of attainable individual benefit, including prevention of HF hospitalizations, treatment optimization, improved quality of life and, potentially, survival, while also informing personalized pre- and peri-implant care.

The delayed survival benefit observed in the patient-level meta-analysis of implantable hemodynamic monitoring effects [9] may help understand why individual disease stage and comorbidity burden matter. In advanced HF, very high event risk may coexist with limited modifiability: prevention of hemodynamic deterioration and hospitalization may not substantially alter short-term prognosis when advanced cardiac disease has limited capacity for structural and functional recovery, and concurrent comorbidities confer additional risk not addressed by PAP-guided management. Conversely, at less advanced stages, sustained hemodynamic stability and prevention of recurrent worsening HF may facilitate reverse cardiac remodeling and systemic recovery and, over time, modify the disease trajectory. Prospective studies are needed to clarify whether and to what extent the treatment of comorbidities adds prognostic benefit in PAP-guided holistic care models.

AI could analyze multidimensional pre- and peri-implant as well as longitudinal datasets to identify nonlinear relationships and interactions that conventional risk assessment may miss. Beyond estimating the risk of adverse clinical outcomes, such models could identify features modifying the response to PAP-guided interventions and support a shift from patient selection based solely on previously established trial inclusion criteria toward the identification of individuals with the greatest potentially achievable benefit from holistic management guided by hemodynamic monitoring, while ensuring equitable and patient-centered decision-making. However, establishing robust models will require generation of representative datasets integrating these characteristics with longitudinal hemodynamics, treatment adjustments, adherence and clinical outcomes, followed by prospective validation.

## Building a Patient-Centered Adaptive Care Cycle

The multidimensional patient characteristics informing eligibility for implantation could also guide post-implant monitoring and treatment (Fig. 1, right). Monitoring intensity does not need to remain constant: prolonged clinical and hemodynamic stability may permit less intensive surveillance, whereas a changing trajectory could trigger temporarily intensified monitoring and more frequent reassessment. Recent analyses of pooled patient-level data showing associations of both absolute PAP levels and longitudinal PAP changes with subsequent HFH and mortality risk [13, 14] further support the prognostic relevance of PAP trajectories. AI could support individualized monitoring by integrating PAP trajectories with other longitudinal clinical information, prioritizing changes by likely clinical relevance, and identifying when current thresholds or monitoring intensity may need adjustment to the patient’s evolving clinical status. For now, clinical responsibility should remain with the healthcare providers, who oversee proposed adaptations and remain the patient’s principal partner in communicating and implementing changes. Digital applications could and should increasingly involve patients and families in surveillance of clinical stability, integrating patient-reported information with hemodynamic monitoring and directing relevant changes to the remote care team. Monitoring could thus be dynamically tailored to individual needs and disease trajectory, enhancing patient satisfaction and, potentially, cost-effectiveness.

Changes in PAP need to prompt more than automatic diuretic escalation. Rising filling pressures may reflect changes in clinical status, related, for example, to acute illnesses such as infections or new-onset atrial fibrillation, worsening kidney function, changes in physical activity, anemia and/or iron deficiency, obstructive pulmonary disease, altered treatment or treatment adherence, dietary changes, or progression of cardiac dysfunction. Identifying and addressing potential causes could enable a more targeted response, including optimization of disease-modifying therapies where appropriate [9, 10]. An adaptive care cycle would thus link monitoring and interpretation with identification and treatment of potential causes, reassessment of treatment response and subsequent adaptation of monitoring and therapies.

Patient participation is integral to this care cycle. Individualized information, education and feedback may improve disease understanding, empower self-management and -responsibility, and support shared decision-making and adherence to recommended treatment changes [17, 18]. Families and other members of the patient’s social network may provide additional support. AI-supported tools could help personalize communication and support according to patient preferences, capabilities, and changing clinical needs. Emerging agentic AI approaches may extend this concept by translating multimodal information into individualized monitoring and treatment support and communicating it in ways adapted to the patient’s health literacy and preferred language [19].

## Equitable Access to AI-Supported Care

Implementation of AI-supported remote care must reduce rather than reinforce existing disparities at both healthcare-system and individual patient levels. Digital access, health literacy and digital literacy, socioeconomic resources, language, cognitive or physical limitations, and family or caregiver support may all impact the ability to participate in remote patient management [17, 18, 20]. Care must also be taken to ensure that AI applications do not perpetuate existing disparities through unrepresentative development datasets or differential performance across patient groups. Equitable implementation requires accessible technologies and infrastructure, patient activation and education, needs-adapted support, and differential alternative care pathways, including non-digital options, for patients unable or unwilling to engage digitally [17, 18, 20]. Healthcare systems must aim to progressively narrow the digital divide, with the principal goal of enabling equitable provision of the right technology and care to the right patient at the right time point.

## Perspectives

As remote patient management guided by implanted hemodynamic monitoring enters reimbursed care, prospective evaluation is needed to determine whether more individualized, AI-supported strategies can further improve its clinical outcomes, workflow efficiency, patient experience, safety, equity and cost-effectiveness. The ongoing PASSPORT-HF trial may provide important evidence in this context. This G-BA-commissioned multicenter trial randomized 559 patients with predominantly NYHA III HF to PAP-guided remote care plus enhanced usual care or enhanced usual care alone within the German healthcare system [21]. Unlike most previous randomized trials evaluating PAP-guided HF management [2–5], extensive serial patient phenotyping in this trial encompasses cardiac structure and function, clinical and laboratory characteristics, comorbidities, mental health and other patient-reported outcomes, adherence and health-economic measures. This comprehensive dataset may help determine how these factors relate to patient risk and clinical outcomes and thus inform future AI-supported models for individualized benefit assessment and a patient-centered adaptive care cycle.

## Data Availability

No datasets were generated or analysed during the current study.
